# Complete Genome Sequence of Two Rift Valley Fever Virus Strains Isolated from Outbreaks in Saudi Arabia (2000) and Kenya (2006 to 2007)

**DOI:** 10.1128/genomeA.00926-16

**Published:** 2016-09-08

**Authors:** Vinay Shivanna, Chester McDowell, William C. Wilson, Juergen A. Richt

**Affiliations:** a Department of Diagnostic Medicine/Pathobiology, College of Veterinary Medicine, Kansas State University, Manhattan, Kansas, USA; b U.S. Department of Agriculture, Agricultural Research Service, Arthropod-Borne Animal Diseases Research Unit, Manhattan, Kansas, USA

## Abstract

The complete genome sequence, including the untranslated regions, of two Rift Valley fever virus (RVFV) strains isolated from mosquitoes that were collected from disease outbreaks in Saudi Arabia (2001) and Kenya (2006 to 2007) were sequenced using next-generation sequencing technology.

## GENOME ANNOUNCEMENT

Rift Valley fever (RVF) is a zoonotic, arthropod-borne, acute, and febrile disease of domestic ruminants endemic to sub-Saharan Africa and the Arabian Peninsula. Epizootics are characterized by abortion, fetal deformities, and significant mortality of young animals (1). RVF virus (RVFV) (family *Bunyaviridae*, genus *Phlebovirus*) is characterized by a tripartite negative-sense, single-stranded RNA genome composed of large (L), medium (M), and small (S) segments (2). In 2000, an outbreak of RVFV affecting humans and livestock was reported in Saudi Arabia and Yemen. This was the first recognized RVFV outbreak outside Africa and Madagascar. The isolate SA01-1322 was obtained from a single pool of *Aedes vexans arabiensis* collected from the outbreak site (3). In 2006/2007, an outbreak of RVF was reported in the Garissa district of Kenya and in Somalia. The Kenya-128B-15 isolate was obtained from *Aedes ochraceus* collected from the outbreak area (4). In the present study, we sequenced the complete genome of these two strains of RVFV to further understand the genetic variation of RVFV isolates.

SA01-1322 and Kenya-128B-15 propagated in C6/36 cells were inactivated in TRIzol-LS reagent (Life Technologies, MD), and RNA was extracted using the RNeasy minikit (Qiagen, CA). Each of the three genome segments was amplified in two parts. The untranslated regions (UTRs) were amplified using a T7 RNA ligase-based strategy described previously (5). Viral RNA and the UTRs were reverse transcribed using SuperScript III first-strand synthesis SuperMix (Invitrogen, CA) and amplified using Platinum PCR SuperMix high fidelity (Invitrogen) with gene-specific primers (primer sequences are available on request). The sequencing library was prepared from 1 ng of DNA for each of the strains using the Nextera XT-DNA library preparation kit (Illumina, CA). Sequencing was performed using standard protocols on the Illumina MiSeq, with 150-bp paired-end reads.

For SA01-1322, the total reads were mapped to reference sequences (accession numbers DQ375401, DQ380197, and DQ380170) using CLC Genomics Workbench (version 7.5.2) and a consensus sequence for the complete L, M, and S segments with average coverages of 7,294×, 5,862×, and 2,323×, respectively, and average coverages of 12,773×, 10,917×, and 4,650×, respectively, for the UTRs were obtained. For Kenya-128B-15, the total reads were similarly mapped to reference sequences (accession numbers EU574010, EU574036, and EU574063), and consensus sequence for the complete L, M, and S segments with average coverages of 7,287×, 4,385×, and 2,235×, respectively, and average coverages of 34,410×, 7,443×, and 32,700×, respectively, for the UTRs were obtained.

A maximum-likelihood phylogenetic tree constructed for each of the L, M, and S segments using MEGA6 showed that the Kenya-128B-15 strain and SA01-1322 strain were most closely related (>99.8%) to strain RVFV-2007002476 (bovine isolate) and strain Saudi 2000-10911 (human isolate), respectively; these strains were isolated from outbreaks in Kenya (2006 to 2007) and Saudi Arabia (2000), respectively. The complete genome sequences (including the UTRs) of the RVFV strains described here contribute to the understanding of the dynamics of RVFV evolution, adaptation, and also to the genetic basis of virulence, since the two characterized RVFV strains showed different phenotypes in sheep (6) and cattle (7).

### Accession number(s).

Complete genomic sequences of L, M, and S segments of RVFV strain Kenya-128B-15 and SA01-1322 have been deposited in GenBank under accession numbers KX096938 to KX096943.
